# What determines the self-rated health of older individuals with stroke compared to other older individuals? A cross-sectional analysis of the Medical Research Council Cognitive Function and Aging Study

**DOI:** 10.1186/1471-2318-13-85

**Published:** 2013-08-22

**Authors:** Nahal Mavaddat, Rianne Van der Linde, George M Savva, Carol Brayne, Jonathan Mant

**Affiliations:** 1 Department of Public Health and Primary Care, University of Cambridge Strangeways Laboratory, Worts Causeway, Cambridge CB1 8RN, UK; 2 Department of Public Health and Primary Care, University of Cambridge Forvie Site, Robinson Way, Cambridge CB2 0SR, UK; 3 School of Nursing Sciences, University of East Anglia Norwich Research Park, Norwich NR4 7TJ, UK

**Keywords:** Stroke self-rated health old age rehabilitation mobility

## Abstract

**Background:**

Poor self-rated health has been associated with poorer objective health outcomes across a range of conditions including stroke. Identification of factors associated with poor self-rated health in stroke survivors has received little attention compared to that in other older individuals. This study identifies determinants of self-rated health in older individuals with or without a history of stroke participating in the population-representative MRC Cognitive Function and Aging Study (MRC CFAS).

**Methods:**

The MRC CFAS is a multicentred longitudinal survey of a population representative sample of people in their 65th year and older at baseline. Baseline interview included questions about functional disability, psychiatric history, independent living status, social interactions, and cognitive function. Multiple logistic regression was used to determine associations between demographic, physical, cognitive, psychological and social factors with poor self-rated health among those with and without stroke.

**Results:**

After excluding those with impaired cognitive function, 776 individuals out of 11,957 reported a stroke. Factors associated with self-rated health were similar between those with or without a stroke in older individuals. Poorer self-rated health in those who had suffered a stroke was associated predominantly with the presence of comorbidity with diabetes (OR 3.5; 95% CI 1.5-8.1) and not “getting out and about” (OR 2.6; 95% CI 1.7-4.1) even after adjustment for disability levels and for depression. In those without a stroke the most important determinants were disability (OR 3.9; 95% CI 3.2-4.8) and not “getting out and about” (OR 2.9; 95% CI 2.5-3.3). The presence of disability was less strongly associated with poor self-rated health in those with a history of stroke than those without due to a substantially higher reporting of poor self-rated health in the non-disabled stroke group than the non-disabled stroke-free group, while those with disabilities reported poor self-rated health irrespective of stroke status.

**Conclusions:**

Self-rated health is determined by a range of psychological and social factors in addition to disability in older patients with stroke. Addressing social integration and mobility out of the home is an important element of rehabilitation for older people with stroke as well as those without.

## Background

As the population in developed nations ages, the burden of stroke, already a cause of considerable disability, [1] is expected to rise [2]. Advances in the management of acute stroke, however, have not been matched by evidence to inform the longer-term support of function, well-being and adaptation of older patients with stroke back into the community [3,4]. Severity, type and location of stroke, access to acute medical and nursing care and stroke units, cognitive impairment, lifestyle factors and presence of co-morbidities have all been related to long-term outcome after a stroke [5,6]. Some evidence however, now suggests a relationship between a stroke survivors’ own subjective health and future outcome. It may, therefore be relevant after stroke to incorporate patient-centred assessments to inform rehabilitation strategies [7-9].

Self-rated health (SRH) is an overall subjective assessment of health involving mental, social and physical dimensions, which correlates well with general well-being [10-12]. It is an independent predictor of poor health outcomes across a range of studies even after adjusting for objective biological measures [13-15]. It has also been shown to predict stroke onset and long-term stroke outcome [9,13]. Addressing elements of daily life that effect subjective well-being are being increasingly recognised as being important to aiding recovery and adaptation to stroke [16-18]. Determinants of subjective health in the general population and in the elderly have been studied widely [11,12,19]. Factors such as marital status, educational level, social class, physical activity, lifestyle, and presence of depression and chronic illness, have all been previously related to self-rated health in elderly populations in differing global settings [20-23]. Few studies, however, have directly compared differences in the factors associated with self-rated health in older populations with stroke to those with no history of stroke. This is potentially important, since understanding self-rated health in stroke survivors can be used to inform rehabilitation services.

The aim of this study is to identify factors that contribute to poor self-rated health in people with stroke in the UK compared with other older individuals in the community. Factors chosen for analysis were those previously related to self-rated health in studies of elderly and normal populations [11,12,19-22]. The study uses data from the MRC Cognitive Function and Aging Study (MRC-CFAS), a study of individuals aged 65 years and over recruited from the community in which physical, psychological, social and cognitive status was determined.

## Methods

The Medical Research Council Cognitive Function and Ageing Study (MRC CFAS) is a multi-centre population based study of individuals aged 65 years and over living in the community, including care homes. The study began in 1991 and was designed to determine the incidence of dementia [24].

The study has six centres across England and Wales chosen to represent the national variation of urban–rural mix, socio-economic deprivation and rates of chronic disease [24]. Five of these centres with identical study designs (Oxford, Nottingham, Newcastle, Cambridgeshire and Gwynedd), are used in the present investigation. The sixth centre (Liverpool) used a different design and is not included. Random samples of people in their 65th year and above were obtained from Family Health Service Authority lists from these five centres. The sample was stratified by age (65–74 years and 75 years and over) and equal numbers were randomly selected from these two age groups with the aim of recruiting 2500 to each centre.

Of those 16258 eligible and available to take part in CFAS, 13004 (80%) agreed to participate. All study centres obtained ethical approval from local research committees and from the Eastern Multicentre Research Ethics Committee Ref: 05/MRE05/37. Eligible participants (or their proxies where appropriate) provided informed consent. Trained interviewers undertook baseline interviews in the participants’ homes.

Socio-demographic factors collected included age, sex, marital status, type of accommodation and social class using the Registrar General’s Occupational Classification [25].

The presence of stroke was determined from self-report through the question: “Have you ever had a stroke that required medical attention?” Time since stroke was determined by subtracting the response to the question: “How old were you when you had the last stroke?” from age at data collection.

General subjective health status or self-rated health (SRH) was determined with the question: “Would you say that for someone of your age, your own health in general is” followed by a list of options from poor to excellent.

Participants were asked about health behaviours including smoking status and alcohol intake. Comorbidities were assessed by the questions: “Have you ever suffered from high blood pressure, angina, heart attack, diabetes or head-injury”?

Functional status was determined by enquiring about activities of daily living (ADL) and instrumental activities of daily living (IADL). ADL disability was defined as requiring help at least several times per week with activities of daily living such as washing, cooking, dressing, or if the respondent was housebound. IADL disability was defined as needing help with heavy housework or shopping and carrying heavy bags.

Cognitive status was determined using the Mini Mental State Examination [26] and the Verbal Fluency Test [27].

History of depression was determined by asking the following questions: “Have you ever consulted a doctor about emotional problems, or problems with your nerves?” followed by “What did the doctor say you had?”

Social variables were assessed by questions including: “Does anyone else live here?”

“How often do you see any of your (children or other) relatives to speak to?”, “Do you have friends in this community?”, “How often do you see any of your neighbours to have a chat or do something with?”, “Do you attend meetings of any community or church or social groups, such as over 60’s clubs, evening classes or anything like that?”, and “In general, do you get out and about as much as you would like to?”

### Analysis

All analyses were performed using Stata 12.0. Any participants who had missing data regarding presence of stroke or SRH or had an MMSE that was missing or less than 18 (as their responses could not be considered reliable for the purposes of this analysis), were excluded from the analyses. The distribution at baseline of demographic, physical, psychological, cognitive and social characteristics were described for participants with and without stroke.

For purposes of logistic regression SRH was initially dichotomised into two groups (good = excellent/good vs. poor = fair/poor). The association between each potential predictor and SRH was assessed using multiple logistic regression. Ordinal and linear regression models were considered in order to increase the power of the analysis, however Brant tests following ordinal logistic regression showed that the proportional odds model was strongly violated by this data (suggesting that linear/ordinal models were not appropriate), although findings based on the linear and logistic models were rarely qualitatively different. Further inspection using different possible cut-points for the dichotomisation of SRH showed that where there were discrepancies between the linear and logistic models, effects were more consistent between the excellent/good vs fair/poor cut-off and the excellent/good/fair vs poor cut off. Either of these dichotomisations would be reasonable choices for estimating the determinants of self-rated health. When using the excellent vs good/fair/poor cut-off, however, estimates of the effects of potential predictors were different in these cases, and may have driven the difference in the linear model. Since we are not concerned with identifying predictors of excellent as opposed to good SRH, logistic regression using the initial cut-point was finally selected as the most robust and informative analysis.

Potential predictors of SRH were defined as follows: Psychosocial variables included age group (65–74, 75–84, and 85+), gender, social class (divided into manual (IIIb, IV, V) and non-manual (I, II, IIIa)), disability (in three groups: no impairment, impairment of IADL only, impairment of ADL), cognition (MMSE divided into four groups, less than 18 or missing, 18–21, 22–25 and 26–30), time since stroke (<1 year, 1–2, 3–5 and >5 years), and presence of depression (yes or no). Prevalence estimates were weighted to adjust for oversampling in the study population of those over 75. Differences in characteristics between participants with and without stroke were calculated using logistic regression adjusting for age and sex.

A multivariate logistic regression model adjusting for demographic, physical, psychological, cognitive and social factors was constructed to explore the association of SRH with stroke. The associations of these factors with SRH in those with and without stroke were then calculated using univariate and multivariate logistic regression. The statistical significance of the differences in the associations between the covariates and SRH for those with stroke compared to those without stroke were calculated by estimating a final logistic regression model for SRH using data from the whole sample and including each covariate and the interaction of each with stroke as independent variables.

## Results

Of 13,004 participants, 1,047 (8.0%) of participants were excluded. These were because participants had one or more of a combination of: no information about stroke history (n=138) or because their MMSE was less than 18 or missing (n=889) or they had missing SRH (n=381). This left 11,957 eligible of whom 11,181 (93.8%) had no history of stroke and 776 (6.2%) who had one or more previous strokes.

Tables 1 and 2 show the frequency of basic demographic, physical, psychological and cognitive profile of older participants reporting a stroke compared to those without a history of stroke. Participants with stroke were older and more likely to be male. After adjusting for age and sex, there were significant differences between people with a history of stroke and those without with regard to several demographic, clinical and psychosocial variables. In particular, there were marked differences in ability to get out and about as much as desired between the two groups, as well as a greater number of people with a history of stroke being depressed and having no friends, and less having contact with neighbours and attending community meetings. More people with a history of stroke reported poor SRH (56.5%) than those without (29.1%). The adjusted odds of having poor SRH in the presence of a history of stroke was over three times (OR 3.1(2.7-3.6)) that in those without a history of stroke.

**Table 1:** Distribution of demographic variables in those with and without stroke in the population of England and Wales aged 65 years and older

	**Individuals with stroke**		**Individuals without stroke**		**P-value**^**†**^
	N	%*	N	%*	
**All**	776	6.2	11,181	93.8	
**Gender**					
Females	400	50.6	6,674	59.0	
Males	376	49.4	4,507	41.0	<0.01
**Age mean**					
Mean	76.2	6.6	74.7	6.6	
**Age group**					
64-74	312	48.8	5,861	60.9	
75-84	375	41.4	4,339	31.9	<0.01
85+	89	9.8	981	7.2	<0.01
**Marital Status categorical**					
Married & Cohabiting	396	52.8	5,782	53.9	
Single	47	6.0	905	7.9	
Widowed	293	35.5	4,079	34.2	
Divorced/separated	40	5.7	414	4.0	
**Type of Accommodation**^‡^					
Independent	741	96.0	11,006	98.7	
Institutionalised	35	4.1	172	1.3	<0.01
**Social Class**					
I Professional	27	3.6	544	5.0	<0.01
II Managerial	179	23.7	2,892	26.5	
IIIa Skilled (non-manual)	85	11.2	1,287	11.7	
IIIb Skilled (manual)	279	36.9	4,076	37.6	
IV Partly skilled	131	17.7	1,606	14.7	
V Unskilled	53	7.0	499	4.6	
**Smoking**					
Never smoked	216	26.7	3,817	33.2	
Current smoker	396	51.6	5,251	47.2	0.01
Former smoker	162	21.7	2,091	19.6	<0.01
**Alcohol intake**					
Never alcohol	98	11.6	1,247	10.7	
Ever alcohol	676	88.4	9,911	89.3	0.16
**Co**-**morbidities**					
High Blood Pressure	402	52.9	3,530	31.8	<0.01
Angina	164	20.8	1,476	13.0	<0.01
Heart Attack	149	19.1	1,093	9.7	<0.01
Diabetes	70	9.2	633	5.5	<0.01
Head Injury	112	14.6	1,254	11.4	<0.01
**Time since stroke**					
<1 year	71	9.2	N/A	N/A	
1-2 years	185	24.6	N/A	N/A	
3-5 years	179	23.4	N/A	N/A	
> 5 years	223	42.8	N/A	N/A	

*percentages backweighted to normal population.

^†^adjusted for age and sex.

^‡^independent - House/Flat/Granny flat, Warden Controlled flat , Institution - Council &Private, Residential home, Nursing Home, Long Hospital Stay.

**Table 2:** **Distribution of physical**, **psychological**, **cognitive and social variables in those with and without stroke in the population of England and Wales aged 65 years and older (Data are count (%) unless otherwise stated)**

	**Individuals with stroke (N=776)**		**Individuals without stroke (N=11,181)**	
	N	%*	N	%*
*Physical*				
**Disabilities**				
None	333	44.9	8,305	76.4
IADL	130	16.4	1,557	13.2
ADL	311	38.8	1,292	10.4
**Self**-**rated Health**				
Excellent	59	7.5	2,349	21.3
Good	280	36.0	5,535	49.7
Fair	319	40.9	2,774	24.4
Poor	118	15.6	523	4.7
*Psychological*				
**Depression**/**Nerves**				
No problems	615	78.4	8,964	79.3
Depression diagnosed	88	12.0	980	9.1
Other diagnosed	70	9.4	1,205	11.3
Not diagnosed, sounds like depression	1	0.1	18	0.2
Not diagnosed, sounds like anxiety	0	0	6	0.05
Not diagnosed, other	1	0.1	3	0.03
**Depression diagnosis**				
No	687	88.0	10,196	90.9
Yes	88	12.0	980	9.1
*Cognition*				
**MMSE**				
26-30	389	52.4	7,559	69.4
22-25	272	33.6	2,788	23.8
18-21	115	14.1	834	6.8
**Verbal Fluency** (**mean**; **s.d.**)	13.9	5.1	16.0	5.4
*Social*				
**Lives alone**^‡^				
With others	468	64.5	6,688	62.6
Alone	272	35.5	4,315	37.4
**Sees children and relatives**^‡^				
Never	12	2.1	109	1.3
Daily	160	26.1	2,290	27.2
2-3 times a week	142	23.1	1,982	23.4
At least weekly	157	25.9	2,099	24.7
At least monthly	67	11.4	1,043	12.3
Less often	70	11.3	971	11.3
**Sees neighbours**^‡^				
Never	33	5.4	299	3.3
Daily	236	39.5	3,972	45.3
2-3 times a week	173	28.8	2,391	27.3
At least weekly	87	14.5	1,379	15.7
At least monthly	36	6.0	399	4.5
Less often	36	5.8	341	3.8
**Has friends**^‡^				
Yes	467	74.8	7,302	81.9
No	161	25.2	1,630	18.1
**Attends meetings**^‡^				
Yes, regularly	232	37.4	3,951	44.7
Yes, occasionally	51	8.4	648	7.3
None	330	54.2	4,296	48.1
**Out and about as much as would like**^‡^				
Yes	362	48.7	8,223	75.2
No	398	51.3	2,908	24.8

*percentages backweighted to normal population.

^†^p values adjusted for age and sex, all p<.01 except living alone and attending meetings p=NS.

^‡^social variables missing for approximately 20% of participants.

Table 3 shows the frequency of physical, psychological and cognitive variables by time since self-reported stroke. Physical disability in the form of ADL and IADL impairment was common in the CFAS population in participants with stroke and persisted: Over half of those who had suffered a stroke more than five years previously reported disability compared to less than a quarter of participants without stroke.

**Table 3:** Distribution of selected variables by time since the event in people with stroke in the population of England and Wales aged 65 years and older

	**Years since stroke**							
	<**1**		**1**-**2**		**3**-**5**		>**5**	
	N	%*	N	%*	N	%*	N	%*
Disability (IADL or ADL)	34	47.0	108	57.3	97	51.4	189	56.8
Depression	7	11.1	26	15.1	25	14.8	28	9.0
Living alone	24	34.0	69	38.4	65	36.1	104	32.6
Seeing relatives less than weekly	15	24.9	38	28.2	36	25.0	56	22.7
Seeing neighbours less than weekly	5	8.6	33	24.8	23	15.3	40	15.9
No friends	11	17.5	38	26.6	46	29.4	62	23.5
Not attending meetings	28	48.4	71	50.8	85	56.0	140	56.9
Not out and about as much as would like to be	38	54.9	101	54.4	94	51.3	154	48.2

*percentages backweighted to normal population.

An additional online table (Additional file 1: Table S1) shows the prevalence and univariate odds ratios whilst Table 4 shows the fully adjusted odds ratios for the effect on ‘poor’ compared to ‘good’ SRH of each factor. The strongest associations of poor SRH in people with stroke were diabetes and “not getting out and about” as much as they wanted. The presence of depression, disabilities and being of lower social class were also significantly independently associated with poor SRH in those with a history of stroke. In people without stroke, the presence of disabilities and “not getting out and about” as much as they wanted were the strongest predictors of poor SRH. Older age was associated with better SRH in both groups.

**Table 4:** **Independent effects of physical**, **psychological**, cognitive and social variables on reporting of poor or fair SRH compared to good or excellent SRH among those with and without stroke in the population of England and Wales aged 65 years and older*

	**Individuals with stroke**		**Individuals without stroke**		**Stroke**:** no stroke**^†^	
	**OR**	**95**% **CI**	**OR**	**95**% **CI**	**OR**	**95**% **CI**
*Socio*-*demographic*						
**Sex**						
Male vs Female	1.2	0.8-1.9	1.1	0.9-1.2	1.1	0.7-1.8
**Age group**						
64-74	1.0	Ref.	1.0	Ref.		
75-84	0.8	0.5-1.2	**0**.**8**	**0**.**7**-**0**.**9**	1.0	0.6-1.5
85+	**0**.**5**	**0**.**2**-**1**.**0**	**0**.**4**	**0**.**3**-**0**.**5**	1.2	0.6-2.8
**Marital status**						
Not married vs currently married	0.6	0.3-1.1	1.0	0.8-1.2	0.6	0.3-1.2
**Social class**						
Manual	**2**.**1**	**1**.**3**-**3**.**2**	**1**.**5**	**1**.**3**-**1**.**7**	1.4	0.9-2.2
*Health behaviour*						
**Smoking**						
Never smoked	1.0	Ref	1.0	Ref	-	-
Former smoker	0.8	0.5-1.4	1.1	0.9-1.2	0.8	0.5-1.3
Current smoker	1.1	0.6-2.0	1.1	0.6-1.3	1.0	0.5-1.9
**Alcohol drinker**						
Ever v Never	1.7	0.9-3.5	**0**.**8**	**0**.**7**-**1**.**0**	**2**.**1**	**1**.**0**-**4**.**3**
*Co*-*morbidities*						
**High Blood Pressure**						
Yes	1.3	0.9-1.9	**1**.**4**	**1**.**3**-**1**.**6**	0.9	0.6-1.4
**Angina**						
Yes	1.4	0.8-2.4	**2**.**0**	**1**.**7**-**2**.**4**	0.7	0.4-1.2
**Heart Attack**						
Yes	1.3	0.8-2.4	**1**.**7**	**1**.**4**-**2**.**0**	0.8	0.4-1.4
**Diabetes**						
Yes	**3**.**5**	**1**.**5**-**8**.**1**	**1**.**7**	**1**.**3**-**2**.**1**	2.1	0.9-5.0
**Head injury**						
Yes	**1**.**7**	**1**.**0**-**3**.**1**	**1**.**3**	**1**.**1**-**1**.**6**	1.3	0.7-2.3
*Physical*						
**Time since stroke**						
<1 year	1.7	0.8-3.3	-			
1-2 years	1.3	0.8-2.2	-			
3-5 years	1.2	0.7-2.0	-			
> 5 years	1.0	Ref.	-			
**Disabilities**						
Not disabled	1.0	Ref.	1.0	**Ref.**		
IADL	1.6	0.9-2.9	3.0	**2**.**6**-**3**.**5**	**0**.**5**	**0**.**3**-**1**.**0**
ADL	**2**.**0**	**1**.**2**-**3**.**4**	**3**.**9**	**3**.**2**-**4**.**8**	**0**.**5**	**0**.**3**-**0**.**9**
*Psychological*						
**Depression diagnosis**						
Yes	**2**.**0**	**1**.**0**-**3**.**8**	**1**.**6**	**1**.**4**-**1**.**9**	1.2	0.6-2.4
*Cognition*						
**MMSE**						
26-30	1.0	Ref.	1.0	Ref.		
22-25	1.2	0.8-2.0	1.0	0.9-1.2	1.2	0.7-2.0
18-21	1.5	0.8-3.0	1.0	0.8-1.3	1.5	0.7-3.0
**Verbal Fluency**						
Per 1 animal named less	**1**.**1**	**1**.**0**-**1**.**1**	**1**.**0**	**1**.**0**-**1**.**1**	1.0	1.0-1.1
*Social*						
**Lives alone**^‡^						
Alone vs living witgh others	1.4	0.7-2.5	1.1	0.9-1.3	1.3	0.7-2.4
**Sees children and relatives**^‡^						
Less than weekly vs weekly or more	1.3	0.8-2.1	1.1	0.9-1.2	1.2	0.7-2.0
**Sees neighbours**^‡^						
Less than weekly vs weekly or more	0.7	0.4-1.2	0.9	0.8-1.1	0.8	0.4-1.3
**Has friends**^‡^						
No	1.1	0.7-1.9	**1**.**3**	**1**.**2**-**1**.**6**	0.8	0.5-1.4
**Attends meetings**^‡^						
None	**1**.**0**	**Ref.**	1.0	Ref.		
Yes, occasionally	**0**.**4**	**0**.**2**-**1**.**0**	0.9	0.7-1.1	0.5	0.2-1.1
Yes, frequently	1.0	0.6-1.5	**0**.**9**	**0**.**8**-**1**.**0**	1.1	0.7-1.7
**Out and about as much as would like**^‡^						
No	**2**.**6**	**1**.**7**-**4**.**1**	**2**.**9**	**2**.**5**-**3**.**3**	0.9	0.6-1.5

*odds ratios and 95% CI’s estimated by multiple logistic regression adjusted for all other variables in the model.

^†^interaction of each independent variable with stroke (i.e. ratio between the effects among those with and without stroke). Statistically significant effects and interactions at p<0.05 are in bold.

^‡^Social variables missing for approximately 20% of participants.

**Figure 1:**
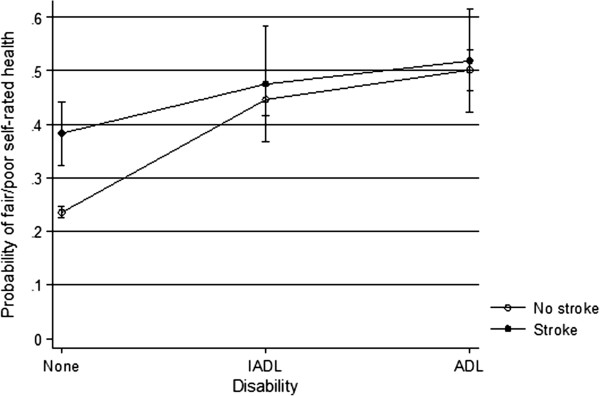
**SRH by disability.** Interaction plot showing the modelled probability of reporting fair or poor self-rated health in those with no disability, IADL or ADL limitations stratified by stroke status. Estimates are standardised for all covariates in the regression model shown in Table 4, and are presented with 95% confidence intervals.

Several of the predictors of poor SRH applied to both people with and without stroke. However, there were some differences in magnitude between the two groups. While ADL disability was associated with poor SRH in both those with and without stroke, this association was twice as strong in those without stroke (OR=3.9; 95% CI=3.2-4.1 in those without stroke compared to OR=2.0; 95% CI=1.2-3.4 in those with stroke). An investigation of the marginal effects (the modelled probabilities of poor SRH by disability and stroke after adjusting for all other covariates) indicates what underlies this difference (Figure 1). In those with stroke, there was a greater probability of reporting poor self-rated health regardless of disability level, such that people with stroke who were not disabled had a much higher probability of reporting poor self-rated health than those without stroke who were not disabled. At the more severely disabled level with ADL impairment, the presence of a stroke made little difference to levels of self-rated health. Angina, high blood pressure and previous heart attack were also more strongly associated with poor SRH in those without stroke than in those with stroke, but the differences as indicated by the interaction terms were not statistically significant. Alcohol consumption, on the other hand, was associated with poorer SRH in people with stroke, and with better SRH in those without stroke. Diabetes had a stronger effect on those with stroke (OR=3.5; 95% CI=3.2-4.8) than those without (OR=1.7; 95% CI: 1.3-2.1), although again the difference between the groups was not statistically significant.

## Discussion

This study of factors associated with self-rated health in individuals with stroke over 65 compared to other older individuals in the population confirms that self-rated health is determined by a broad range of factors - demographic, physical, social and psychological - and suggests that these are similar in older people with or without a history of stroke. ‘Not getting out and about’ and the presence of disability or impairments of daily living were strongly associated with poor self-rated health in both those with and without a history of stroke. The association of poor self-rated health with disability was however significantly greater in people without stroke. In both those with and without a stroke, older age was associated with better comparative self-rated health, and the presence of comorbidities with poorer subjective health. The strong association between diabetes and poor self-rated health in those with stroke, of particular importance in the context of rising rates of comorbidity between diabetes and stroke [28], could relate to the additional difficulties in self-care with diabetes that may arise in the context of physical and cognitive impairments associated with having suffered a stroke. For example, the potential need to self-administer insulin in the face of physical disability, the increased cognitive demands of taking multiple medications, the reduced ability to participate in physical activity, and greater difficulty in accessing medical care and specialised diabetes clinics, may all contribute to worsened diabetic control and potentially subjective health in those with stroke. The presence of diabetes and stroke in combination has been previously shown to be strongly associated with a higher risk of disabilities, poor subjective health and mortality in elderly populations [29]. Other studies suggest that diabetes in association with other chronic medical conditions reduces health-related quality of life in an additive fashion [30]. Depression and being of manual social class, both associated with self-rated health in the general population [31,32] and with poorer functional recovery from stroke [33-36] were also strongly related to poor self-rated health in older individuals, with or without a history of stroke.

Degrees of physical functional impairment or disability have previously been reported as one of the strongest factors associated with poor self-rated health in the general and older population and in patients with stroke [9,12]. In our study, however, the presence of physical disability was less strongly associated with poor self-rated health in those with a history of stroke than those without. This is due to a substantially higher rate of reporting of poor self-rated health in the non-disabled stroke group than the non-disabled stroke-free group, while those with ADL or IADL limitations report poor SRH irrespective of stroke status. Literature on the association of disability with self-rated health suggests that those with chronic disabilities may place greater emphasis on factors other than their physical functioning when reporting self-rated health [37]. Indeed in patients with stroke, other factors were associated more strongly with poor self-rated health than physical disability. For example, the presence of depression in patients with stroke, independent of physical disability and any cognitive impairment, was as strongly associated with poor self-rated health as disability levels, confirming suggestions from other studies that depression may be of equal or even greater importance than physical disability as a predictor of subjective wellbeing and quality of life after stroke [18,38].

An important finding of our study was that poor self-rated health independent of the presence of functional disability and depression, in both those with and without stroke, was strongly associated with a negative response to the question of whether participants were “getting out and about” as much as they would like to. However, in our study no statistically significant independent association between self-rated health was found with more objective social factors such as living alone, or having friends or seeing neighbours. It is likely that the way individuals respond to the “out and about” question reflects to some degree levels of perceived satisfaction with social isolation or interaction. More than five years after a stroke, nearly half of older people with a history of stroke in the CFAS study felt they were not getting out and about as much as they would like to compared with one quarter of those who had never had a stroke. At one to two years after stroke, one of the peak times for social isolation according to our study, a quarter of patients with stroke were seeing neighbours less than weekly compared to just over ten percent of those without, and over a quarter had no friends although there was no difference in amount of family contact between those with and without stroke.

The role of social isolation and its converse, participation in social activities and presence of social support and networks is being increasingly confirmed as being an independent factor in stroke outcomes. A number of studies suggest that social dimensions may be of central importance to health related quality of life and physical recovery from stroke [39-42]. However, not “getting out and about” may be influenced by factors other than lack of physical mobility, depression and social interaction, and may reflect to some degree internal qualities such as confidence, motivation and expectations of recovery either positive or negative [43,44]. Trials such as the “Getting Out Of The House” study [44] and the “Out and About” trial [45] focussing on outdoor mobility designed to help patients to get out of the house after stroke are therefore particularly pertinent. Such trials aim to assess ways of assisting the individual to spend more time outside the home such as by helping to overcome patients’ personal barriers to leaving the house, as well as setting mobility goals and delivering interventions such as practicing driving with patients, or helping them to use the bus or to walk local routes [44]. Our findings suggest that similar initiatives might have promise if applied to older individuals in the population more generally. Any attempts at improving accessibility and friendliness of the local environment such as improvements in local transportation and shop access should also aid the older population and those with disabilities to “get out and about”, potentially improving their quality of life and subjective health.

### Strengths and limitations of the study

This study has several strengths including the large size of the cohort of older participants studied with a history stroke, as well as the comparisons made with a large control group of other older people in the community without stroke. Most long-term studies of patients after stroke have either not compared findings with control groups or have used comparison with population norms [3]. Our study reported findings by time since stroke and addressed the impact of time after a stroke with self-rated health, an advantage since most studies of post-stroke patients lack control for duration of stroke onset [41,46]. The limitations of the study include data being collected retrospectively and dependent upon self-report in an older population. Although more contemporary than other studies, the CFAS was carried out in the 1990s and changes in disease patterns, treatments and outcomes may have occurred since then. The study was cross-sectional, so a causal relationship between poor self-rated health and associated factors cannot be concluded. For example, not getting out of the house may lead to poorer self-rated health, but it may be that poor self-rated health may lead to decreased likelihood of getting out of the house. As with all observational studies, there is the risk that our associations are confounded by further variables that were not included. For example, ‘frailty’ was not taken into account, and it is plausible that this has further independent effects that were not adjusted for in our model (which included adjustment for activities of daily living, but not frailty per se).

## Conclusions

In conclusion, our findings emphasise the need to attend to psychosocial aspects of wellbeing as well as addressing physical functioning in both people who have had stroke and those who have not. This reinforces the suggestion that many different types of interventions involving multidisciplinary care may be required to effectively rehabilitate patients with stroke [18,47,48]. In particular, efforts at improving subjective wellbeing should include attempts at social reintegration of patients after stroke into the community and getting patients “out and about”.

## Competing interests

All authors declared that they have no competing interests.

## Authors’ contributions

CB is Principal Investigator of the MRC CFAS Study. NM, RV and GS contributed to analysis of data. All authors contributed to study design and intellectual input. All authors read and approved the final manuscript.

## Authors’ information

CB is Professor of Public Health, JM is Professor of Primary Care Research and NM is Clinical Lecturer in Primary Care Research at the University of Cambridge. GS is Senior Lecturer in Nursing Sciences at the University of East Anglia. RV is currently a PhD student at the University of Cambridge.

## Pre-publication history

The pre-publication history for this paper can be accessed here:

http://www.biomedcentral.com/1471-2318/13/85/prepub

## Supplementary Material

Additional file 1: Table S1 Online Table: Prevalence and univariate odds ratios for poor/fair self-rated health by studied variables in the population of England and Wales aged 65 years and older with or without stroke *. Click here for file
