# Supplementary material for: What determines the self-rated health of older individuals with stroke compared to other older individuals? A cross-sectional analysis of the Medical Research Council Cognitive Function and Aging Study
Source: BMC Geriatr. 2013 Aug 22;13:85. doi: 10.1186/1471-2318-13-85 (PMC3847649; doi:10.1186/1471-2318-13-85)
Supplement: Additional file 1: Table S1 — Online Table: Prevalence and univariate odds ratios for poor/fair self-rated health by studied variables in the population of England and Wales aged 65 years and older with or without stroke *. [file 1471-2318-13-85-S1.docx]

**Additional Online Table: Prevalence and univariate odds ratios for poor/fair self-rated health by studied variables in the population of England and Wales aged 65 years and older with or without stroke ***

|  | **Individuals with Stroke (n=776)** | | | | | | **Individuals without stroke (n=11181)** | | | | | |
| --- | --- | --- | --- | --- | --- | --- | --- | --- | --- | --- | --- | --- |
|  | **Excellent**  **/Good** | | **Fair/**  **Poor** | | **OR**  **95% CI** | | **Excellent**  **/Good** | | **Fair/**  **Poor** | | **OR**  **95% CI** | |
|  | **N %** | | **N %** | |  | | **N %** | | **N %** | |  | |
|  | 339 | 43.6 | 437 | 56.4 |  |  | 7 ,884 | 71.0 | 3,297 | 29.0 |  |  |
| *Socio-demographic* |  | |  |  |  |  |  |  |  |  |  |  |
| **Sex** |  |  |  |  |  |  |  |  |  |  |  |  |
| Female | 178 | 45.1 | 222 | 54.9 | 1.0 |  | 4,622 | 69.7 | 2,052 | 30.3 | 1.0 |  |
| Male | 161 | 42.0 | 215 | 58.0 | 1.1 | 0.8-1.4 | 3,262 | 72.7 | 1,245 | 27.3 | 0.9 | 0.8-0.9 |
| **Age group** |  |  |  |  |  |  |  |  |  |  |  |  |
| 64-74 | 132 | 42.3 | 180 | 57.7 | 1.0 |  | 4,273 | 72.9 | 1,588 | 27.1 | 1.0 |  |
| 75-84 | 163 | 43.7 | 212 | 56.3 | 1.0 | 0.7-1.3 | 2,935 | 67.8 | 1,404 | 32.3 | 1.3 | 1.2-1.4 |
| 85+ | 44 | 49.4 | 45 | 50.6 | 0.8 | 0.5-1.2 | 676 | 69.0 | 305 | 31.0 | 1.2 | 1.0-1.4 |
| **Marital status** |  |  |  |  |  |  |  |  |  |  |  |  |
| Married | 173 | 44.3 | 218 | 55.7 | 1.0 |  | 4,191 | 73.2 | 1,560 | 26.8 | 1.0 |  |
| Not married | 166 | 42.8 | 219 | 57.2 | 1.0 | 0.8-1.4 | 3,692 | 68.3 | 1,736 | 31.7 | 1.3 | 1.3-1.4 |
| **Institution** |  |  |  |  |  |  |  |  |  |  |  |  |
| Independent | 326 | 43.8 | 415 | 56.2 | 1.0 |  | 7,781 | 71.1 | 3,225 | 28.9 | 1.0 |  |
| Institutionalised | 13 | 38.8 | 22 | 61.3 | 1.3 | 0.7-2.7 | 100 | 57.5 | 72 | 42.5 | 1.7 | 1.3-2.3 |
| **Social class** |  |  |  |  |  |  |  |  |  |  |  |  |
| Non-manual | 166 | 56.7 | 125 | 43.3 | 1.0 |  | 3,617 | 77.1 | 1,106 | 23.0 | 1.0 |  |
| Manual | 166 | 35.9 | 297 | 64.1 | 2.4 | 1.8-3.2 | 4,075 | 66.4 | 2,106 | 33.6 | 1.7 | 1.6-1.8 |
| *Health behaviour* |  |  |  |  |  |  |  |  |  |  |  |  |
| **Smoking** |  |  |  |  |  |  |  |  |  |  |  |  |
| Never smoked | 95 | 44.4 | 121 | 55.6 | 1.0 |  | 2,751 | 72.8 | 1,066 | 27.2 | 1.0 |  |
| Former smoker | 181 | 45.8 | 215 | 54.2 | 0.9 | 0.7-1.3 | 3,695 | 70.7 | 1,556 | 29.3 | 1.1 | 1.0-1.2 |
| Current smoker | 63 | 37.8 | 99 | 62.2 | 1.2 | 0.8-1.9 | 1,431 | 68.9 | 660 | 31.1 | 1.2 | 1.0-1.4 |
| **Alcohol drinker** |  |  |  |  |  |  |  |  |  |  |  |  |
| Never | 42 | 42.1 | 56 | 58.0 | 1.0 |  | 807 | 65.1 | 440 | 34.9 | 1.0 |  |
| Ever | 297 | 43.9 | 379 | 56.1 | 1.0 | 0.6-1.5 | 7,068 | 71.7 | 2,843 | 28.3 | 0.7 | 0.7-0.8 |
| *Co-morbidities* |  |  |  |  |  |  |  |  |  |  |  |  |
| **High Blood Pressure** |  | |  |  |  |  |  |  |  |  |  |  |
| No | 165 | 43.8 | 208 | 56.2 | 1.0 |  | 5,622 | 74.1 | 2,024 | 25.9 | 1.0 |  |
| Yes | 174 | 43.5 | 228 | 56.5 | 1.0 | 0.8-1.4 | 2,258 | 64.2 | 1,272 | 35.8 | 1.6 | 1.4-1.7 |
| **Angina** |  |  |  |  |  |  |  |  |  |  |  |  |
| No | 286 | 46.6 | 326 | 53.4 | 1.0 |  | 7,159 | 74.3 | 2,543 | 25.8 | 1.0 |  |
| Yes | 53 | 32.1 | 111 | 67.9 | 1.8 | 1.3-2.6 | 723 | 48.9 | 753 | 51.1 | 2.9 | 2.6-3.3 |
| **Heart Attack** |  |  |  |  |  |  |  |  |  |  |  |  |
| No | 291 | 46.3 | 336 | 53.7 | 1.0 |  | 7,351 | 73.3 | 2,733 | 26.7 | 1.0 |  |
| Yes | 48 | 31.9 | 101 | 68.1 | 1.8 | 1.2-2.7 | 531 | 49.0 | 562 | 51.0 | 2.8 | 2.5-3.2 |
| **Diabetes** |  |  |  |  |  |  |  |  |  |  |  |  |
| No | 321 | 45.4 | 385 | 54.6 | 1.0 |  | 7,545 | 72.0 | 3,000 | 28.0 | 1.0 |  |
| Yes | 18 | 25.5 | 52 | 74.5 | 2.4 | 1.4-4.2 | 339 | 54.1 | 294 | 46.0 | 2.2 | 1.9-2.6 |
| **Head injury** |  |  |  |  |  |  |  |  |  |  |  |  |
| No | 299 | 44.8 | 365 | 55.2 | 1.0 |  | 7,046 | 71.5 | 2,875 | 28.5 | 1.0 |  |
| Yes | 40 | 36.3 | 72 | 63.7 | 1.5 | 1.0-2.2 | 835 | 66.7 | 419 | 33.3 | 1.2 | 1.1-1.4 |
| *Physical* |  |  |  |  |  |  |  |  |  |  |  |  |
| **Disabilities N(%)** |  |  |  |  |  |  |  |  |  |  |  |  |
| None | 194 | 58.3 | 139 | 41.7 | 1.0 |  | 6,609 | 79.6 | 1,696 | 20.4 | 1.0 |  |
| IADL | 49 | 35.3 | 81 | 64.7 | 2.3 | 1.5-3.5 | 763 | 47.4 | 794 | 52.6 | 4.1 | 3.6-4.5 |
| ADL | 95 | 30.0 | 216 | 70.0 | 3.2 | 2.3-4.4 | 503 | 38.2 | 789 | 61.8 | 6.1 | 5.4-6.9 |
| **Time since stroke** |  |  |  |  |  |  |  |  |  |  |  |  |
| > 5 years | 146 | 44.3 | 177 | 55.7 | 1.0 |  |  |  |  |  |  |  |
| 3-5years | 79 | 44.8 | 100 | 55.2 | 1.0 | 0.7-1.5 |  |  |  |  |  |  |
| 1-3 years | 79 | 42.5 | 106 | 57.5 | 1.1 | 0.8-1.6 |  |  |  |  |  |  |
| <1 year | 29 | 42.3 | 42 | 57.7 | 1.2 | 0.7-2.0 |  |  |  |  |  |  |
| *Psychological* |  |  |  |  |  |  |  |  |  |  |  |  |
| **Depression diagnosis** |  |  |  |  |  |  |  |  |  |  |  |  |
| No | 311 | 45.1 | 376 | 54.9 | 1.0 |  | 7,297 | 72.0 | 2,899 | 28.0 | 1.0 |  |
| Yes | 28 | 32.7 | 60 | 67.4 | 1.8 | 1.1-2.8 | 585 | 60.3 | 395 | 39.7 | 1.7 | 1.5-1.9 |
| *Cognition* |  |  |  |  |  |  |  |  |  |  |  |  |
| **MMSE** |  |  |  |  |  |  |  |  |  |  |  |  |
| 26-30 | 200 | 51.0 | 189 | 49.0 | 1.0 |  | 5,593 | 74.3 | 1,966 | 25.7 | 1.0 |  |
| 22-25 | 99 | 36.1 | 173 | 63.9 | 1.8 | 1.3-2.5 | 1,796 | 64.4 | 992 | 35.6 | 1.6 | 1.4-1.7 |
| 18-21 | 40 | 34.0 | 75 | 66.0 | 2.0 | 1.3-3.1 | 495 | 59.4 | 339 | 40.6 | 1.9 | 1.7-2.3 |
| **Verbal Fluency^†^** |  |  |  |  |  |  |  |  |  |  |  |  |
| Mean (SD) | 15.1 | 5.3 | 12.9 | 4.8 | 1.1 | 1.1-1.1 | 16.6 | 5.4 | 14.7 | 5.1 | 1.1 | 1.1-1.1 |
| *Social* |  |  |  |  |  |  |  |  |  |  |  |  |
| **Lives alone** ^‡^ |  |  |  |  |  |  |  |  |  |  |  |  |
| With others | 214 | 45.1 | 254 | 54.9 | 1.0 |  | 4,824 | 72.5 | 1,864 | 27.5 | 1.0 |  |
| Alone | 112 | 41.4 | 160 | 58.6 | 1.2 | 0.9-1.6 | 2,957 | 68.9 | 1,358 | 31.1 | 1.2 | 1.1-1.3 |
| **Sees children and relatives**^‡^ |  |  |  |  |  |  |  |  |  |  |  |  |
| At least weekly | 199 | 43.1 | 260 | 56.9 | 1.0. |  | 4,503 | 71.0 | 1,868 | 29.0 | 1.0 |  |
| Less than weekly | 69 | 46.1 | 80 | 54.0 | 0.9 | 0.6-1.3 | 1,538 | 72.8 | 585 | 27.2 | 0.9 | 0.8-1.0 |
| **Sees neighbours**^‡^ |  |  |  |  |  |  |  |  |  |  |  |  |
| At least weekly | 220 | 44.0 | 276 | 56.0 | 1.0 |  | 5,572 | 72.3 | 2,170 | 27.7 | 1.0 |  |
| Less than weekly | 45 | 42.9 | 60 | 57.1 | 1.1 | 0.7-1.6 | 675 | 62.3 | 364 | 34.8 | 1.4 | 1.2-1.6 |
| **Has friends** ^‡^ |  |  |  |  |  |  |  |  |  |  |  |  |
| Yes | 218 | 46.2 | 249 | 53.8 | 1.0 |  | 5,338 | 73.3 | 1,964 | 26.7 | 1.0 |  |
| No | 59 | 37.0 | 102 | 63.0 | 1.5 | 1.0-2.2 | 999 | 61.9 | 631 | 38.1 | 1.7 | 1.5-1.9 |
| **Attends meetings** ^‡^ |  |  |  |  |  |  |  |  |  |  |  |  |
| Yes, regularly | 108 | 46.5 | 124 | 53.6 | 1.0 |  | 2,994 | 76.1 | 957 | 23.9 | 1.0 |  |
| Yes, occasionally | 28 | 52.6 | 23 | 47.4 | 0.7 | 0.4-1.3 | 473 | 73.3 | 175 | 26.7 | 1.2 | 1.0-1.4 |
| None | 133 | 40.2 | 197 | 59.8 | 1.3 | 0.9-1.8 | 2,852 | 66.6 | 175 | 33.4 | 1.6 | 1.4-1.7 |
| Out and about as much as would like ^‡^ | | |  |  |  |  |  |  |  |  |  |  |
| Yes | 210 | 57.8 | 152 | 42.2 | 1.0 |  | 6,520 | 79.3 | 1,703 | 20.7 | 1.0 |  |
| No | 121 | 29.8 | 277 | 70.2 | 3.2 | 2.3-4.3 | 1,340 | 46.0 | 1,568 | 54.0 | 4.5 | 4.1-4.9 |

*****percentages backweighted to population

**^†^**per one animal named less

^‡^ social variables missing for approximately 20% of participants
